# Cogito: automated and generic comparison of annotated genomic intervals

**DOI:** 10.1186/s12859-022-04853-1

**Published:** 2022-08-04

**Authors:** Annika Bürger, Martin Dugas

**Affiliations:** 1 Institute of Medical Informatics, Westfälische Wilhelms-Universität Münster, Albert-Schweitzer-Campus 1, 48149 Münster, Germany; 2 Institute of Medical Informatics, Heidelberg University Hospital, Seminarstr. 2, 69117 Heidelberg, Germany

**Keywords:** Correlation, Statistics, Genomic interval, Reproducible data analysis, Data integration

## Abstract

**Background:**

Genetic and epigenetic biological studies often combine different types of experiments and multiple conditions. While the corresponding raw and processed data are made available through specialized public databases, the processed files are usually limited to a specific research question. Hence, they are unsuitable for an unbiased, systematic overview of a complex dataset. However, possible combinations of different sample types and conditions grow exponentially with the amount of sample types and conditions. Therefore the risk to miss a correlation or to overrate an identified correlation should be mitigated in a complex dataset. Since reanalysis of a full study is rarely a viable option, new methods are needed to address these issues systematically, reliably, reproducibly and efficiently.

**Results:**

Cogito “COmpare annotated Genomic Intervals TOol” provides a workflow for an unbiased, structured overview and systematic analysis of complex genomic datasets consisting of different data types (e.g. RNA-seq, ChIP-seq) and conditions. Cogito is able to visualize valuable key information of genomic or epigenomic interval-based data, thereby providing a straightforward analysis approach for comparing different conditions. It supports getting an unbiased impression of a dataset and developing an appropriate analysis strategy for it. In addition to a text-based report, Cogito offers a fully customizable report as a starting point for further in-depth investigation.

**Conclusions:**

Cogito implements a novel approach to facilitate high-level overview analyses of complex datasets, and offers additional insights into the data without the need for a full, time-consuming reanalysis. The R/Bioconductor package is freely available at https://bioconductor.org/packages/release/bioc/html/Cogito.html, a comprehensive documentation with detailed descriptions and reproducible examples is included.

**Supplementary Information:**

The online version contains supplementary material available at 10.1186/s12859-022-04853-1.

## Background

With the ongoing success of next-generation sequencing data (NGS), the amount of available genomic data is constantly growing. Lists of annotated genomic intervals, i. e. data tables with genomic positions and attached values from complex biological settings, have to be analyzed to gain insight into specific scientific questions. Relevant data types that can be represented as intervals include ChIP-seq peak regions with attached score values, RNA-seq expression values for various genes, information about mutations from DNA-seq data, data regarding the methylation status of CpGs, as well as results of other types of experiments. In the following text all this information attached to the plain genomic intervals will be referred to as *attached values*.

Often, two or more data types are examined for two or more sample conditions within one NGS study. A popular example in cancer research includes the combination of ChIP-seq and RNA-seq data, and the use of tumor material and healthy cells as conditions. As investigating one specific research question often requires sophisticated data analysis, scanning the whole complexity of the collected data in full detail may be neglected due to the required time and effort. Unknown or unsuspected dependencies in the data might therefore remain undiscovered. Public data repositories like the Gene Expression Omnibus (GEO) [1] provide valuable raw data, but lack a comprehensive overview of data correlations and interdependencies.

Scientific projects of multiple experimental approaches and conditions typically include the following workflow regarding bioinformatic analyses: After producing data in the laboratory, a quality control of the data assures that it can be used for sophisticated analyses. After this, biologists/physicians and bioinformaticians develop a common understanding and overview of the present data and agree on an analysis strategy, which then—often in an iterative process—will be executed. The trend towards growing data size and multiomics settings causes a combinational explosion of possibilities how to look at a multimodal dataset, only bivariate combinations of samples grow exponentially with the number of samples. This makes it essential to have a shared understanding and overview of the whole dataset to avoid time-consuming analyses with little outcome on the one hand and on the other hand not to miss potential interesting interdependencies.

A number of bioinformatics tools for the comparison of lists of genomic intervals already exists. LOLA [2], which is available as a Bioconductor package in R, works with multiple technologies and concentrates on statistics about overlaps between user-supplied sets of genomic intervals and region sets of a defined reference database. The recently published web-based tool epiCOLOC [3] provides the comparison of the uploaded dataset to a broad collection of epigenomic data. Both LOLA and epiCOLOC can also compare user-defined genomic tracks to tracks from reference databases.

Other tools focus on statistical comparisons between user-defined genomic tracks; they use different mathematical background models and provide a broad range of output statistics and graphics. Among these, StereoGene [4] concentrates on different measures of correlation (i.e. the kernel correlation) of two tracks consisting of continuous data. It computes different aspects of kernel-based correlation statistics and visualizes these interdependencies. GAT (Genomic Association Test) [5] was published in 2013 as a python script. The algorithms allows users to compare several genomic tracks of interest to several reference tracks, with correlation values based on a simulation approach. Genome Track Analyzer [6] is a web based tool where users can compare two genomic tracks with each other with several statistical methods. KLTepigenome [7] is published as a collection of R scripts to investigate correlation between several epigenomic tracks, the mathematical background bases on functional principal components, and the Karhunen-Loève transform. The method was introduced for the analysis of ChIP-seq data, but can be used for other epigenomic datasets as well. GenometriCorr [8] is provided as R package and performs statistical tests regarding spatial correlation of a reference and a query dataset of interval data. It produces extensive tables with corresponding plots. The Genomic Hyperbrowser [9] is an extensive web-based tool, which allows registered users to perform different analyses, calculate statistics and to generate plots about datasets of genomic tracks. BedSect [10] produces plots of interval overlaps and can be used from a web-based interface. The web-based meta tool Coloc-stats [11] does not directly compare interval-based data, but allows the combination of results from several other tools mentioned above.

Despite the multitude of algorithms already available for the comparison of genomic intervals, a flexible and user-friendly program to generate comprehensive and customizable reports about automated comparisons of genomic ranges with attached values is still lacking.

To support an understanding and a general overview over complex datasets and defining a suitable analysis strategy, we developed Cogito, the “COmpare annotated Genomic Intervals TOol”. This novel workflow allows to combine and analyze the output of multiple laboratory techniques and conditions, requires little user interaction, and offers an elaborate and comprehensive output report that helps to reveal novel findings and to generate hypotheses for further investigation.

## Implementation

**Fig. 1:**
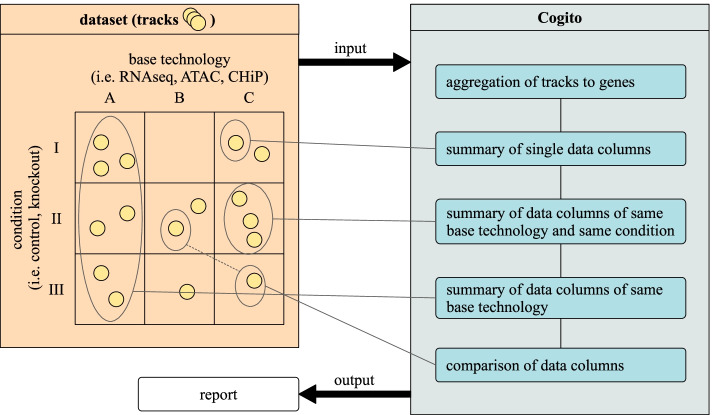
Workflow of Cogito. After preparation and aggregation of the input data (tracks) on gene level, Cogito summarizes and compares all provided data columns for single tracks and groups of tracks and creates a comprehensive output report

Cogito is implemented in R [12], which is widely used in bioinformatics projects. The tool follows the R/Bioconductor quality standards, and can easily be integrated in existing workflows. Figure 1 shows the general workflow of Cogito, as outlined below.

### Input

Cogito can handle any genomic or epigenomic data obtained in a biological experiment that can be represented as interval-based data. One sample consisting of intervals with optionally attached values for each interval is here referred to as *track*. For use with Cogito, all tracks have to be provided as GRanges data objects which can be easily obtained from BED, CSV, tab-separated text files etc. For the latter, metadata columns containing attached values (e.g. fold changes for ChIP-seq peaks) are explicitly allowed. The laboratory base technology (e.g. ChIP-seq or RNA-seq) of every sample, as well as the underlying condition (e.g. control or knockout), has to be provided either through the use of variables in the main functions of the R package, or in a supplied configuration file.

In addition to files or data objects containing the tracks which have to be analyzed, Cogito requires information about the locations of the genes in the corresponding genome or the specification of a reference genome which should be used to extract this information from public data bases.

### Data preparation

Prior to actual data processing, Cogito performs various steps of data preparation.

At the beginning, formal tests for consistency are employed to ensure that all provided data is of the right format and belongs to the same reference genome.

Next, all tracks with attached values are aggregated to gene level with regards to the chosen reference genome. In this process, each interval of each track is assigned to the closest gene within a predefined maximum distance. Thus, several cases can occur: First, if there is more than one interval of one track assigned to the same gene, the attached values—if there are any—of these intervals will be aggregated depending of there underlying condition (e.g. the maximum of the height of two ChIP-seq peaks or the mean of two RNA-seq expression values). Second, if there is no interval assigned to a gene, the value of this gene for the corresponding track is set to “not-defined”. Third, if no values are attached to a track, the number of intervals of this track which are assigned to a gene will be stored. Optionally, the user can specify to aggregate the provided tracks to a custom set of ranges (i.e. a set of enhancers) instead of genes.

Hence, the final result of this aggregation is one single table per dataset or analysis, where each row corresponds to one gene, and each column to an attached value column of a track, or a track without a respective attached value.

### Analysis and visualization of the track data

The central and defining feature of Cogito is its summarizing and reporting functionality. With the help of the aggregated table resulting from the preparation step, several plots and summarizing tables are generated.

In the first part of the analysis, each track (i.e. column of the aggregated table) is summarized individually. The scale of the respective values is determined as either numerical, i.e. rational or interval scaled, or categorical, i.e. ordinal, nominal or binary. Depending on this scale, a location parameter is computed for each track, e.g. the mean value for data on a rational scale, and a dispersion parameter like the standard deviation for interval scale data. Additionally, each track is visualized through an appropriate plot. Table 1 shows which parameters are calculated according to the underlying scale of attached values to the intervals of tracks.

**Table 1:** Overview of scales for interval-attached values. An example, localization and dispersion parameters, as well as visualization method are provided

Scale	Example	Localization	Dispersion	Visualization
Binary	Mutation yes or no	Most frequent value	Both values present?	Barplot
Nominal	Category of mutation	Most frequent value	No. of present values	Barplot
Ordinal	Level of methylation	Median	Quantiles	Ordered barplot
interval	Scores of ChIP-seq peaks	Arithmetic mean	Standard deviation	Boxplot
Rational	Expression value	Geo. mean	Coefficient of variation	Boxplot

In the second part of the analysis, groups of attached values are summarized and plotted instead of individual columns. These groups can either consist of tracks sharing the base technology and condition, tracks sharing base technology with different conditions, or user defined groups. For example: a specific subgroup of ChIP-seq tracks regarding histone modifications shall be analyzed. This step does not only summarize the data, but also provides basic quality control regarding group-wise tendencies. The relation of several columns of attached values compared among each other can provide insight into noticeable quality differences in the data, possible batch effects, or the presence or absence of knock-out effects.

Finally, different columns of attached values are compared pairwise with each other. Depending on their respective scales, each pair is compared statistically and visualized by an appropriate plot. Table 2 presents the respective statistical tests. Cogito does not perform any tests on absolute heights, but concentrates on tests which are independent from scaling effects like normalization.

**Table 2:** Statistical tests for pairwise comparison of interval-attached values. For several combinations, more than one test is applicable

Scale	Binary/nominal/ordinal	Interval/rational
Binary/nominal/ordinal	Pearson’s Chi-squared test/Fishers exact test	Wilcox or Kruskal–Wallis Test/ rank sum test/t-test
Interval/rational	Wilcox or Kruskal–Wallis test/rank sum test/t-test	Correlation test

While this approach results in at least $n^2/2$ sets of characteristic numbers, correlations, and associated plots for *n* samples, this complexity and computational cost is nevertheless essential for an unbiased analysis and the discovery of potentially hidden links and relationships between tracks.

### Output

The output of Cogito’s default workflow, as outlined in the steps above, is one single, comprehensive report, which contains a rich set of information and visualizations. This report is provided either as portable document format (PDF) or as hypertext markup language document (HTML). In addition, the R Markdown file [13–15] is provided, which is the basis of the report. RData files of the processed data and a settings file in json format are prepared as complementary output. If users are interested in a more generic overview of the data, they can use the provided PDF or HTML report. Users with interest in customizing or further developing the report can subsequently continue to extend the produced Markdown file in combination with the processed data stored in the RData file. To change settings or parameters users can edit the settings file and rerun the analysis.

## Results

In general, many NGS-based medical and biological projects include a multitude of possible study setups, which address different scientific questions and come with their own unique challenges. In many cases, however, there is a common general setup with samples of different conditions, which are subsequently investigated through several base technologies such as RNA- or DNA-sequencing. Some studies contain many conditions with a more limited number of samples in each condition, while others focus on fewer conditions (e.g. wildtype and a knock-out or tumor condition), but include many samples.

Hence, we chose two structural different example datasets of real preprocessed sequencing data to show the wide applicability of Cogito.

### First example dataset

The first dataset consists of murine data published by King et al. [16]. It provides expression values from RNA-seq, methylation data and ChIP-seq data for each one or two samples of up to 9 conditions.

King et al. examined the effects of DNA methylation in murine embryonic stem cells (ESCs) on histone modifications H3K4me3, H3K27me3, and H3K27ac [16]. Briefly, the wildtype condition (J1) was compared to triple knock-out mice (TKO), double knock-out mice (DKO) and mice with reintroduced methylation status (conditions TKO3a1, TKO3a2, TKO3b1, DKO3a1, DKO3a2 and DKO3b1).

The preprocessing of the dataset is described in the appendix.

An overview of the murine example dataset is shown in Table 3.

**Table 3:** Overview of example murine ESC dataset from King et al. [16]

	Base technology					
	mRNA	ChIP	ChIP	ChIP	ChIP	RRBS
		H3K4me1	H3K4me3	H3K27ac	H3K27me3	
*Condition*						
J1	1	1	1	1	2	2
TKO	1	1	1	1	2	2
TKO3a1	2	1		2	1	2
TKO3a2	2	1	1	2	1	2
TKO3b1	1	1	1	1	1	2
DKO	1	1		2	1	1
DKO3a1	1	1		1	1	1
DKO3a2	1	1		1	1	1
DKO3b1	1	1		1	1	1

This dataset includes samples of up to 9 conditions (J1, TKO, TKO3a1, TKO3a2, TKO3b1, DKO, DKO3a1, DKO3a2, DKO3b1), which were processed with three different base technologies: gene expression by RNA-seq, transcription factor binding sites by ChIP-seq, and methylation status by RRBS

Within Cogito gene expression in reads per kilo base per million mapped reads (RPKM) from RNA-seq and Homer ChIP-seq peak scores were interpreted as rational values. To accommodate the bimodal distribution of the methylation data, it was binned into the categories *low*, *medium* and *high*, depending on the fraction of methylated cytosine for a CpG context (below $20\%$, between $20\%$ and $80\%$, or above $80\%$); consequently, the data was interpreted as ordinal scaled values.

**Fig. 2:**
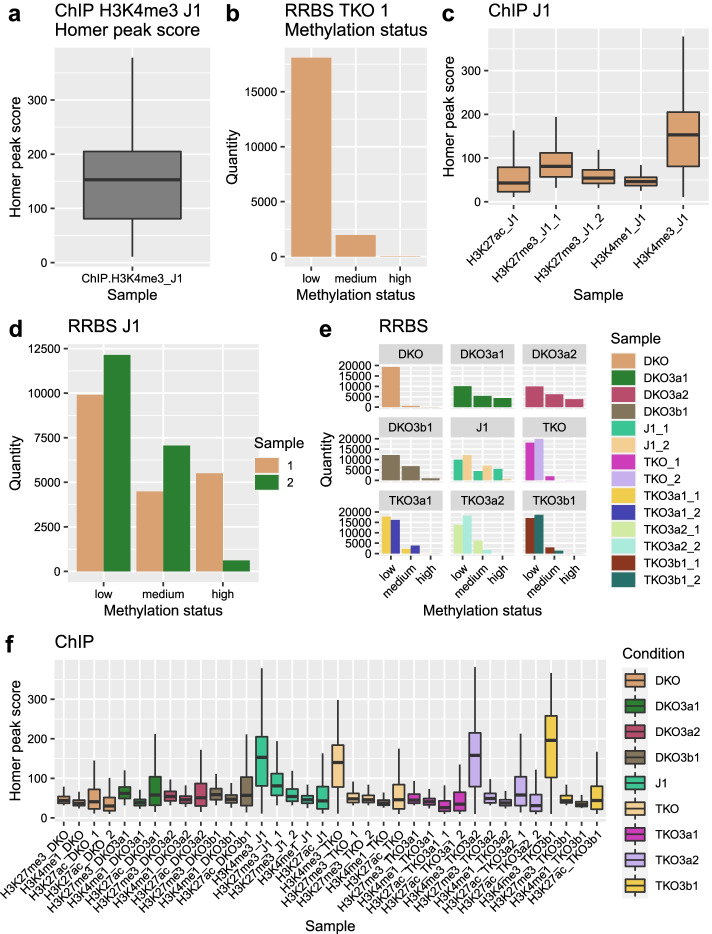
Cogito base output for King et al.’s murine dataset tracks and subgroups of tracks. **a** ChIP-seq peak score visualization of a single track (interval attribute). **b** Methylation status plot for a single track (ordinal attribute). **c** ChIP-seq score overview for replicate wildtype samples (condition J1). **d** Barplot depiction of RRBS replicates for condition J1. **e** Methylation status plot per track, grouped by condition. **f** ChIP-seq scores per track, color-coded by condition

After this initial data preparation, the standard workflow of Cogito was executed for the murine dataset: In the first step, all individual tracks were summarized using a suitable mean value and dispersion function, and visualized with their associated scale’s default visualization routine. For the rational values of the gene expression and ChIP-seq peak score data, the geometric mean and the coefficient of variation were calculated, and a boxplot was chosen as representative visualization. For the ordinal methylation value the median was used as Cogito’s mean value and quantiles as dispersion index, along with an ordered barplot as graphical representation. This step resulted in one overview table of all samples, with their respective means and dispersion values, and one characteristic figure per sample (example see Fig. 2a, b).

In the next step, all tracks with the same base technology and condition were combined to groups, and corresponding group-wise plots were created, e.g. all performed ChIP-seq experiments of the healthy control group were integrated into one plot. Since these plots directly depend on the groups present in the respective dataset, their total number may vary considerably between datasets of otherwise similar size and sample number. For the dataset of King et al., replicates of RNA-expression data are provided; exemplary group plots for RNA-seq condition TKO3a1 are shown in Fig. 2c, d.

Subsequently, all tracks of one base technology were summarized based on their respective means and dispersion values, and then displayed in one plot. With RNA-seq, ChIP-seq and methylation data present, three technology-specific plots were created, as shown exemplarily in Fig. 2e, f.

**Fig. 3:**
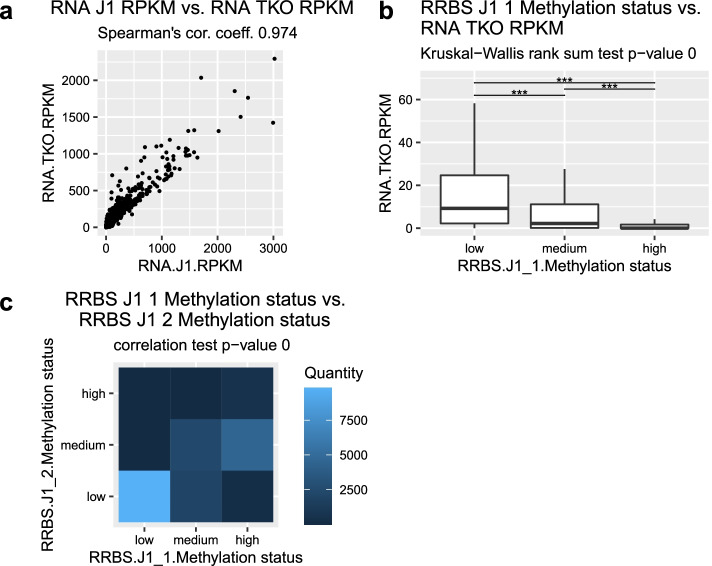
Advanced Cogito output graphics for pairwise comparisons in King et al.’s dataset. **a** Comparison plot for the gene expression of two tracks. **b** Correspondence visualization of the methylation status of one track and the gene expression of another track. **c** Correlation heatmap of the methylation status of two tracks: the lighter the color is, the higher is the quantity of genes which have the corresponding methylation status

**Fig. 4:**
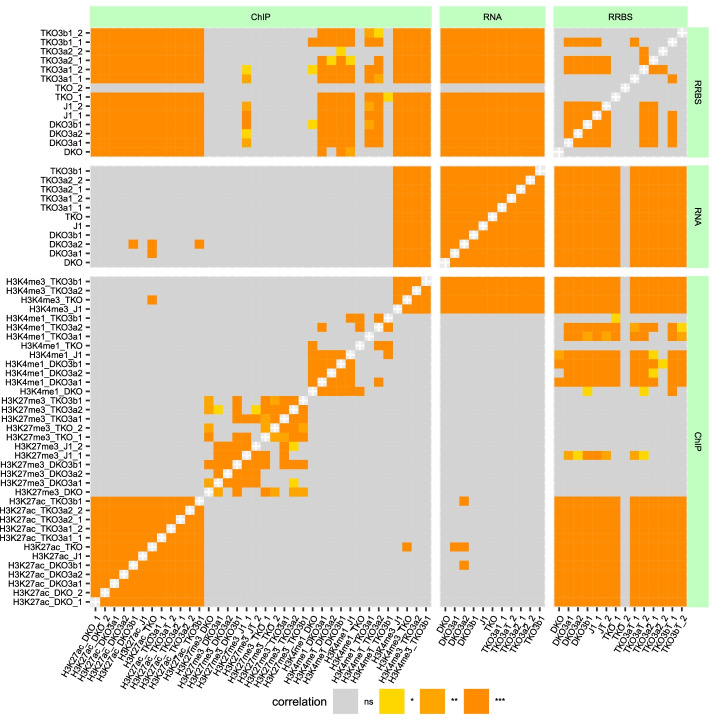
Overview correlation heatmap for the full murine sample set of King et al. A high-level visualization of pairwise comparisons of all samples contained in the murine example dataset presents rich information density in one heatmap, and emphasizes possible connections

In the last step of the workflow, Cogito was used to compare all single tracks with all other tracks, regardless of their scale. These comparisons were then visually summarized in a high-level heatmap, as shown in Fig. 4, and an associated table. For each meaningful comparison (i.e. each of the compared samples contains more than one value) with a significant statistical test (defined as corrected p-value of performed correlation test $\le 0.1$ per default), one plot was created, the p-value is rounded to three digits so p-values $<0.0005$ are displayed as 0. A set of example plots is shown in Fig. 3.

With the given complexity of $n=3$ included base technologies and up to $c=9$ conditions, the murine dataset of King et al. is relatively large and contains a high amount of information. While a sophisticated and customized analysis is needed to uncover the less obvious interconnections and dependencies hidden in the data, Cogito’s high-level overview analysis succeeds in replicating key results, and emphasizes central features of the dataset. Figure 2e clearly shows that the methylation levels in knock-down conditions TKO and DKO are lower than in the wildtype (J1), but almost restored to wildtype level in samples where Dnmts were reintroduced (sample DKO3a1 etc.). Furthermore, Cogito’s sample pair plots and overview correlation heatmap both indicate that the gene expression is widely preserved in all samples and conditions, as shown in Figs. 3a and 4.

### Second example dataset

The second, human dataset was taken from the pediatric T-cell lymphoblastic lymphoma (T-LBL) project of Khanam et al. [17]. The dataset provides copy number variants, mutation information from DNA-seq, and methylation data from up to 16 samples in two conditions.

Khanam et al. identified molecular markers of prognostic relevance in heterogeneous lymphoblasts by systematic integration of information regarding CNVs, gene mutations, and methylation status data of different patients at two points of time, namely primary tumors at diagnosis (TP), and tumors after relapse (TR).

The preprocessing of the dataset is described in the appendix.

Table 4 shows an overview of the samples with their data types and associated condition.

**Table 4:** Overview of the human T-LBL dataset from Khanam et al

	Base technology		
	CNV	Methylation	DNA
*Condition*			
TP (primary)	16	15	14
TR (relapse)	6	5	6

This human dataset includes up to 16 tracks in two conditions TP (primary timepoint) and TR (relapse timepoint) examined by three base technologies: Copy Number Variants (CNV), methylation status and mutations (DNA-sequencing)

The methylation data was preprocessed similarly to the first murine example dataset. Due to its essentially bimodal distribution the signal was binned into three ordered categories from low, over medium, to high methylation scores. Copy number variants were interpreted as an ordinal attribute with categories 0 (deletion/del), 1 (del), 2 (loss of heterozygosity LOH), 3 (duplication/dup) and 4 (dup). Since the preprocessed mutation information consists of data regarding the presence or absence of a variant at a position, it was interpreted as a binary attribute, i.e. analog to a nominal scaled attribute with only two possible values.

The workflow of Cogito was executed with default parameters. In the first step of the analysis, all individual tracks were summarized by calculating an appropriate mean and dispersion value. A corresponding visualization was produced to allow for an objective impression of the data (exemplarily shown in Additional file 1: Fig. S1a, and b). The human dataset consists of a binary (i.e. nominal) attribute, representing whether or not a gene shows a specific mutation, and two ordinal valued attributes, methylation status and the copy number variants. Consequently, the median was chosen as Cogito’s location parameter for the ordinal values, and the most frequent value was calculated in the case of the binary value. The number of different or used values, respectively, was taken as dispersion parameter.

In the next step of the Cogito workflow, all tracks with shared base technology and condition were grouped together, and a summarizing visualization was generated for these groups. As the human dataset from Khanam et al. contains up to 16 tracks for a single group, the generated plots were more complex than those shown for the previous murine example dataset. An example visualization is shown in Additional file 1: Fig. S1c, d.

Subsequently, all tracks with the same base technology were combined and visualized as barplots. Example plots are shown in Additional file 1: Fig. S1e, f.

In the last step of the workflow, Cogito was used to compare all possible track pair combinations. The resulting overview was exported as a table and as a heatmap. In addition, one plot per pair-wise comparison was produced, and a corresponding statistical test result was reported. Two exemplary comparison heatmaps are shown in Additional file 2: Fig. S2.

Khanam et al. deeply investigated the dataset, while Cogito provides an overview analysis which is not intended as a substitute for a deep custom analysis in complex datasets. Nevertheless, its aggregation functionality and visualization routines provide useful and accurate summaries of the data. Cogito’s summary of mutation numbers indicates that sample TR_14 of the relapse condition shows hypermutation and sample TR_15 also shows an increase in the number of mutations, as shown in Additional file 1: Fig. S1e. In contrast, the methylation status of all samples is similar even across conditions (i.e. Additional file 2: Fig. S2a). These trends, overview plots and figures match with the general observations of Khanam et al. paper.

## Discussion

We developed the tool Cogito, the COmpare annotated Genomic Intervals TOol, to summarize, integrate and compare tracks of genomic intervals attached with additional data values and collected with different laboratory techniques in one single, reproducible and comprehensive report. Executed after the quality control of a given dataset it can help to get an overview and a common understanding of the data and to develop a strategy for further, deeper investigation. As mentioned above Cogito is implemented as a R/Bioconductor package that follows the R/Bioconductor standard, and thus can be executed under Windows, Linux, and macOS. The availability as R/Bioconductor package simplifies its maintenance routine and installation process. This makes it easy to be used by laboratory staff and medical professionals with basic knowledge in computer science and in R, but also by computer scientists to get an overview over their dataset and as a starting point for custom analyses. Furthermore, it allows a local installation of the program, so that it is possible to use the tool in projects with e.g. personal data of patients or confidential datasets. Moreover, tools with local installation options have notable advantages over any web-based tool in case of larger datasets, which are typically impractical to upload to a server-based system. Cogito includes a comprehensive and detailed documentation that also allows non-specialists to work through the standard workflow of the tool without the need of extensive user interaction. Its generic approach allows the analysis of cross-platform NGS data types and other interval-based genomic information. With its customizable report function, Cogito’s analysis is intended as general starting point for a project-specific analysis of the input data. Cogito combines an unbiased overview with a wide applicability in multi-condition and multi-sample datasets. We demonstrated the versatility of Cogito on two published datasets, showed its ability to generate hypotheses based on correlation values, and presented a subsumption of its overview analysis with the original studies’ results.

We contrasted Cogito’s functionality to those of other tools with equivalent objectives (Table 5), and contextualized differences and similarities. As the scope and usage of the tools is rather diverse, we did not conduct purely quantitative comparisons of the algorithm’s run-time or similar, but concentrated on qualitative contrasts. A catalog of criteria was used for this purpose, which we categorized into the following aspects: required and possible input, performed analysis, resulting output and general aspects.

The selection of tools for this comparison was based on the algorithms referred to in StereoGene, which was extended by follow ups of these tools and other more recently published algorithms.

The program IntervalStats [18] was excluded due to lack of availability of its code; the more generic software BEDTools [19] and GenomicRanges [20] were not included further because of their more general, infrastructure-related scope and general requirements of expert knowledge from the user, which are thus not directly comparable to Cogito.

Among the more comparable tools with Cogito, the algorithms GenometriCorr, Genome Track Analyzer, KLTepigenome, Genomic Hyperbrowser, GAT, StereoGene and BedSect concentrate on the comparison of two or more user defined tracks of genomic intervals with different statistical methods, while LOLA and epiCOLOC also take reference tracks of databases into account. They all differ in the amount of required user input and output data. The tools Genomic Hyperbrowser, BedSect and epiCOLOC do not offer a local installation, which is a critical requirement for the analysis of highly confident patient data.

**Table 5:** Qualitative comparison of Cogito to other tools. The rows show different qualitative criteria, the columns indicate the considered tools

Program features		GenometriCorr [8]	Genome track analyzer [6]	KLTepigenome [7]	Genomic hyperbrowser [9]	GAT [5]	StereoGene [4]	BedSect [10]	LOLA [2]	epiCOLOC [3]	Cogito
Input	Cross platform analysis	+	+	+	+	+	+	+	+	+	+
	Supports multiple ($\ge 2$) conditions	$-$	$-$	+	+	+	$-$	+	+	+	+
	Little user interaction required	+	$-$	$-$	$-$	+	+	$-$	+	+	+
Analysis	Works with attached values	$-$	$-$	$-$	$-$	$-$	$-$	$-$	$-$	$-$	+
	Automatic systematic investigation	$-$	$-$	$-$	$-$	+	+	+	+	$-$	+
Output	Produces readable report	+	$-$	$-$	$-$	$-$	$-$	$-$	$-$	$-$	+
	Output further adaptable	$-$	$-$	$-$	$-$	$-$	+	$-$	$-$	$-$	+
General	Local installation available	+	+	+	$-$	+	+	$-$	+	$-$	+

A $+$ shows a tool matching the criteria, a − the lack of that function. If a cell of the matrix is empty, the category was not applicable for that specific tool

Notably, most of the tools in the qualitative comparison do not provide a output report which can then serve as starting point for further investigation. Furthermore, they all require more specific user interactions to create a chosen statistical result, as well as specific, limited plots about particular aspects of the data. Cogito, on the other hand, produces a comprehensive report with a detailed analysis of the supplied genomic intervals, while respecting the types of additional annotations and requiring only little user interaction. At the same time, Cogito allows for optional, extensive customization of its report by expert users. The strength of the general approach of Cogito is at the same time also its limitation. Thus, Cogito does not serve as a dedicated analysis tool for any deep investigations of specific scientific questions, but gives an overview for any supplied genomic intervals. Another limitation of Cogito is the underlying aggregation of the genomic intervals to genes. This simplifies the interpretation of the results and is usually applicable for genomic data, but may be misleading for data which is not directly referring to the gene structure of the genome.

## Conclusions

Analyzing complex genomic datasets in an unbiased way is a major computational and logistical challenge, however this work is essential for a comprehensive data analysis. Hence, we introduced the novel R/Bioconductor package Cogito, a tool that allows to manage different types of genomic intervals, so-called “tracks”, and which can analyze differently scaled values that are attached to these tracks. Considering two examples of real datasets, we have demonstrated that Cogito can smoothly integrate and analyze differently structured data. With the easy-to-use standard workflow supplied by Cogito, only limited user interaction is required to create an elaborate and comprehensive output report for revealing novel findings and generating hypotheses for further investigation. Cogito can be customized by more experienced users in order to adapt the output reports to specialized settings. This combination of functions sets the Cogito package apart from other algorithms that conduct comparisons of interval-based data. Cogito can provide valuable analyses for biologists and computer scientists alike. Cogito is available as an R/Bioconductor package and can therefore be easily included in bioinformatics workflows. With the ongoing success of next-generation sequencing and the ever-increasing data treasure stored in public repositories, we believe that unbiased, automated, easy-to-use analysis systems like Cogito will gain importance in the future.

## Availability and requirements

*Project name*: Cogito “COmpare annotated Genomic Intervals TOol”.

*Project home page*: https://www.bioconductor.org/packages/release/bioc/html/Cogito.html.

*Operating systems*: Platform independent.

*Programming language*: R.

*Other requirements*: R 4.1 or higher.

*License*: LGPL-3.

*Any restrictions to use by non-academics*: None.

### Supplementary Information

**Additional file1**.** Fig. S1**: Cogito output of the human dataset from Khanam et al. (a) Methylation status visualization, depicted as a barplot. (b) CNV overview plot for one track (ordinal scaled attribute). (c) Boxplot group visualization for RRBS-tracks and condition TR. (d) Boxplot group visualization for DNA-tracks with condition TR. (e) Presence or absence of mutations in DNA samples, split by condition. (f) Methylation status of all tracks, grouped by condition.

**Additional file2**.** Fig. S2**: Example comparison plots for the human dataset of Khanam et al. (a) Comparison between the methylation status of two samples. Colors indicate the quantity of genes with the specified attached values. (b) Correlation between CNVs in two samples of different conditions.

## Data Availability

The R/Bioconductor-package Cogito, its source code, detailed documentation including a manual and a vignette with examples and exemplary data are freely available at https://www.bioconductor.org/packages/release/bioc/html/Cogito.html (license: LGPL-3). The murine data from King et al. that support the evaluation of the presented software are available in NCBI GEO database [21] under the accession number GSE77004. The human epigenetic data that served as example dataset are available in the European Nucleotide Archive [24] under accession number PRJEB36436, methylation array data and SNP array data are available under the accession numbers E-MTAB-8762, E-MTAB-9382, and E-MTAB-8763.

## Appendix

### Preprocessing of murine example data

The murine data from King et al. was downloaded from the NCBI GEO database [21] under the accession number GSE77004. The available ChIP-seq data (GSE77002) was processed as described in [16]: After an alignment with bowtie [22] with parameters selecting for uniquely mapped, best-matching reads and a maximum of two mismatches per read, the peak calling was done with the Homer software suite’s findPeaks [23] algorithm and an input control. Subsequently, the raw peaks were filtered with the following parameters: -F 8 for H3K4me3, -size 1000 -minDist 3000 -F 4 -tagThreshold 32 for H3K27me3, -F 4 for H3K27ac and -size 1000 -minDist 1000 -nfr for H3K4me1.

The available RNA-seq RPKM values per gene from the same study, provided under the accession number GSE77003, were utilized directly. The methylation status, measured by RRBS, was similarly taken from the published files (accession number GSE84103), which contain the fraction of methylated cytosine for every CpG context supported by a minimum of 5 reads. To reduce the complexity and better balance the dataset of the murine example data, four samples (3x TFX and 1x mut) were removed before the following examination.

### Preprocessing of human example data

The raw data of this dataset is available in the European Nucleotide Archive (ENA) (https://www.ebi.ac.uk) [24] under accession number PRJEB36436, methylation array data and SNP array data are available under the accession numbers E-MTAB-8762, E-MTAB-9382, and E-MTAB-8763. All raw data was preprocessed as described in [17].
