# Supplementary figures and images for: Cogito: automated and generic comparison of annotated genomic intervals
Source: BMC Bioinformatics. 2022 Aug 4;23:315. doi: 10.1186/s12859-022-04853-1 (PMC9351259; doi:10.1186/s12859-022-04853-1)

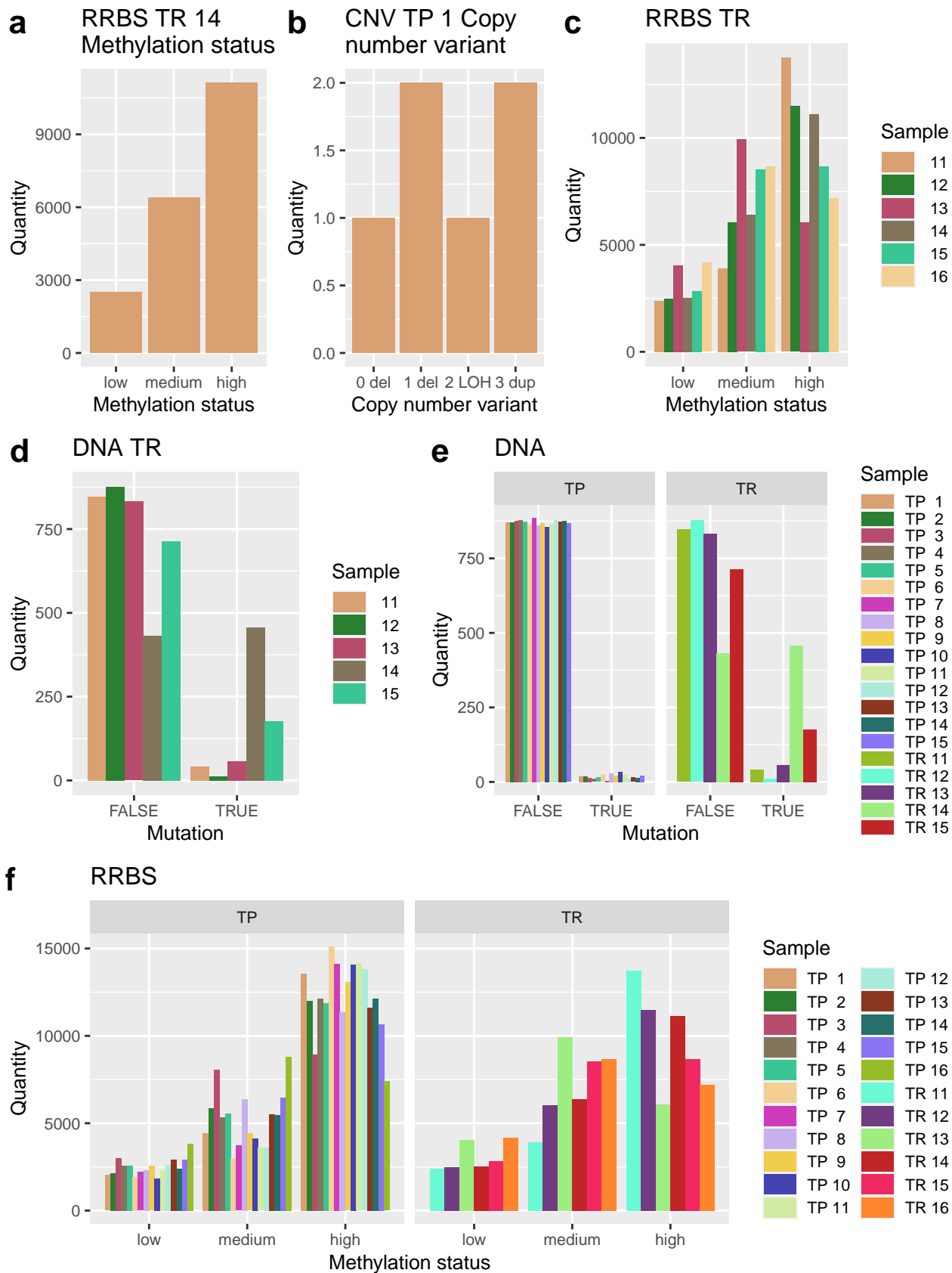

Supplement: Supplementary file 1 — Additional file1. Fig. S1: Cogito output of the human dataset from Khanam et al. (a) Methylation status visualization, depicted as a barplot. (b) CNV overview plot for one track (ordinal scaled attribute). (c) Boxplot group visualization for RRBS-tracks and condition TR. (d) Boxplot group visualization for DNA-tracks with condition TR. (e) Presence or absence of mutations in DNA samples, split by condition. (f) Methylation status of all tracks, grouped by condition. [file 12859_2022_4853_MOESM1_ESM.pdf]
