# Supplementary material for: Cogito: automated and generic comparison of annotated genomic intervals
Source: BMC Bioinformatics. 2022 Aug 4;23:315. doi: 10.1186/s12859-022-04853-1 (PMC9351259; doi:10.1186/s12859-022-04853-1)
Supplement: Supplementary file 2 — Additional file2. Fig. S2: Example comparison plots for the human dataset of Khanam et al. (a) Comparison between the methylation status of two samples. Colors indicate the quantity of genes with the specified attached values. (b) Correlation between CNVs in two samples of different conditions. [file 12859_2022_4853_MOESM2_ESM.pdf]

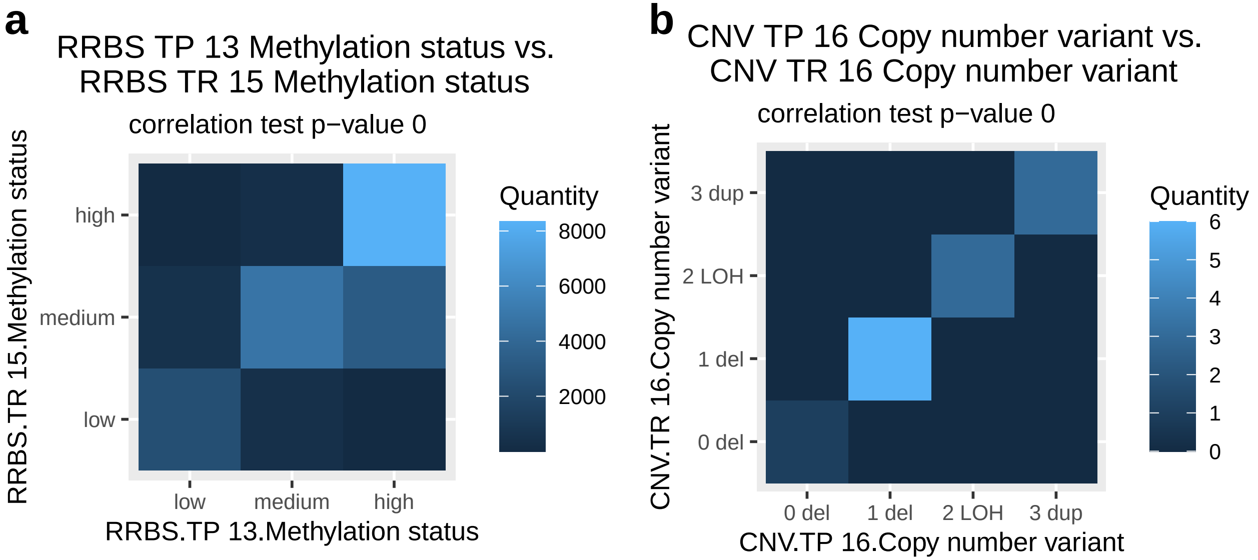
a
RRBS TP 13 Methylation status vs.
RRBS TR 15 Methylation status
correlation test p−value 0
RRBS.TR 15.Methylation status
high
medium
low
low
medium
high
RRBS.TP 13.Methylation status
Quantity
8000
6000
4000
2000
b
CNV TP 16 Copy number variant vs.
CNV TR 16 Copy number variant
correlation test p−value 0
CNV.TR 16.Copy number variant
3 dup
2 LOH
1 del
0 del
0 del
1 del
2 LOH
3 dup
CNV.TP 16.Copy number variant
Quantity
6
5
4
3
2
1
0
